# Supplementary material for: High spontaneous integration rates of end-modified linear DNAs upon mammalian cell transfection
Source: Sci Rep. 2023 Apr 26;13:6835. doi: 10.1038/s41598-023-33862-0 (PMC10133325; doi:10.1038/s41598-023-33862-0)
Supplement: Supplementary file 1 — Supplementary Information. [file 41598_2023_33862_MOESM1_ESM.pdf]

High spontaneous integration rates of end-modified linear DNAs upon mammalian cell transfection

Supporting Information

Samuel Lim*[1,2], R. Rogers Yocum[3], Pamela A Silver[1,2], Jeffrey C Way*[3]

Author affiliations

1. Department of Systems Biology, Harvard Medical School, Boston, Massachusetts 02115, United States.
2. Wyss Institute for Biologically Inspired Engineering, Boston, Massachusetts 02115, United States.
3. General Biologics, Inc., 108 Fayerweather Street, Unit 2, Cambridge, Massachusetts 02138, United States.

Author contacts

Samuel Lim: samuel_lim@hms.harvard.edu

R. Rogers Yocum: rog.yocum@genbiologics.com

Jeffrey C Way: jeff.way@genbiologics.com

Pamela A Silver: pamela_silver@hms.harvard.edu

*Corresponding authors

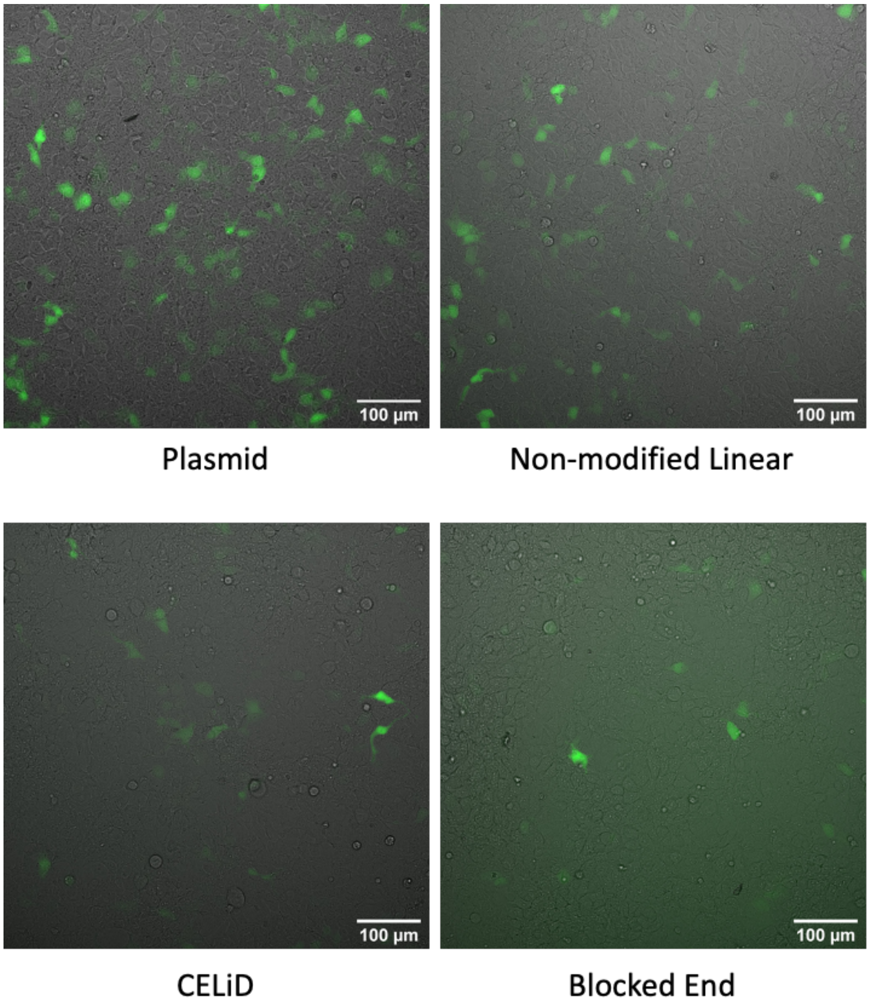

Figure S1. Images of the HEK293 cells transfected with each type of DNA construct 24 hours after transfection. Images taken from brightfield and green fluorescent channel were overlapped. Scale bar indicates 100 μm.

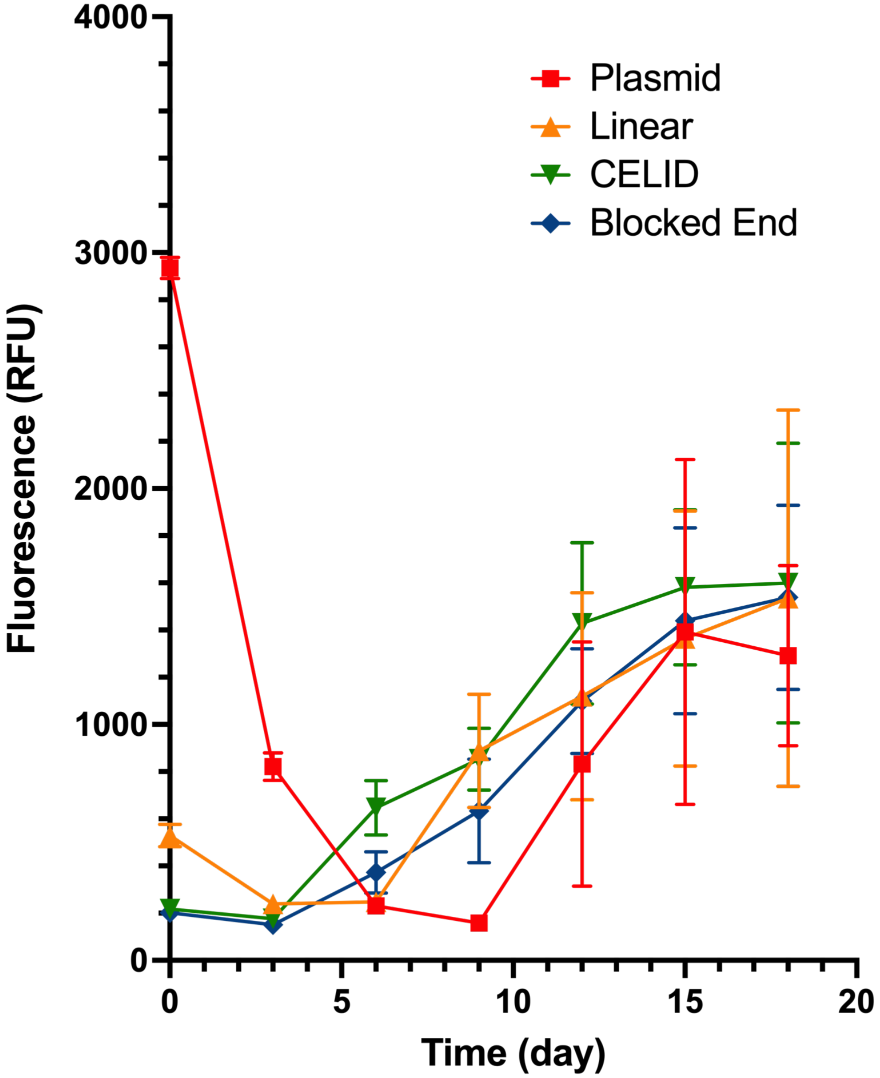

Figure S2. Change in fluorescence intensity of transfected cells over time. Median fluorescence intensity of the GFP positive cell population was measured from day 0 to 21, using flow cytometry. In the cells transfected with plasmid and linear DNAs, the cellular expression levels in GFP+ cells increases significantly when these cells become a stabilized fraction of the total. This result suggests that the integration events occur in chromosomal loci that promote particularly strong expression.

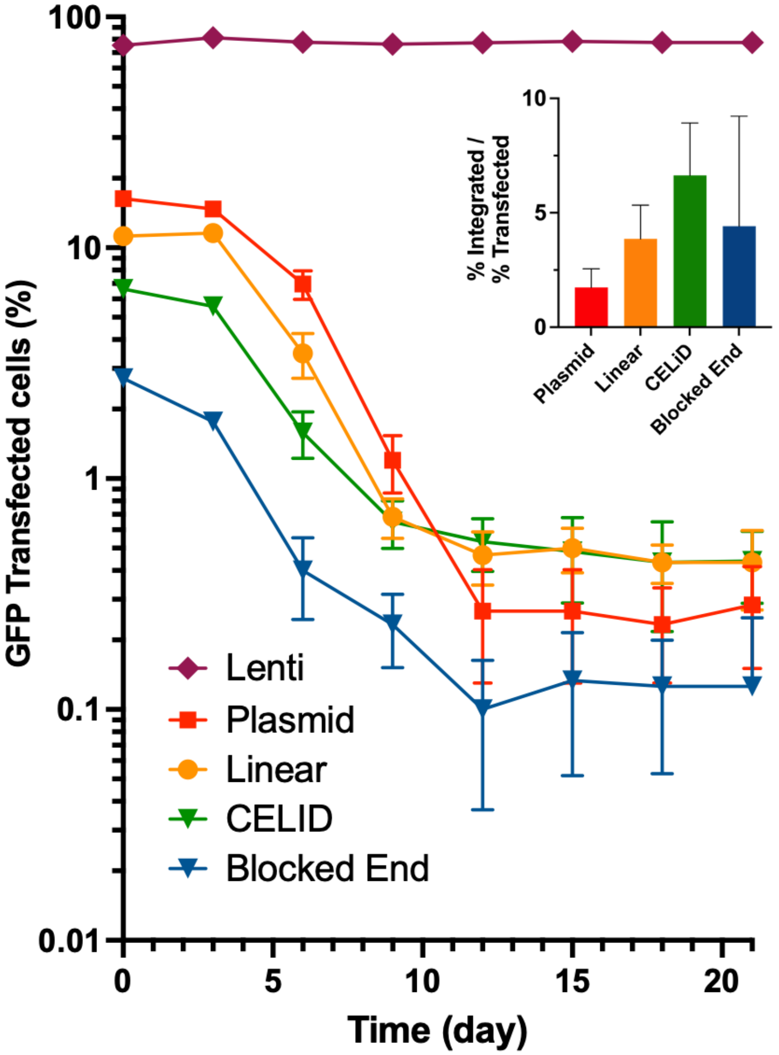

Figure S3. A biological repeat of the DNA transfection experiment. Results were consistent with those described in Figure 3. Additionally, a lentiviral delivery system with a vector containing the GFP reporter was used as a positive control for stable integration.

```
LOCUS       OQ117120                6450 bp    DNA     circular SYN 28-JAN-2023
DEFINITION  Vector PAS_122122, complete sequence.
ACCESSION   OQ117120
VERSION     OQ117120.1
KEYWORDS    .
SOURCE      Vector PAS_122122
  ORGANISM  Vector PAS_122122
            other sequences; artificial sequences; vectors.
REFERENCE   1  (bases 1 to 6450)
  AUTHORS   Lim,S., Yocum,R.R., Silver,P.A. and Way,J.C.
  TITLE     High spontaneous integration rates of end-modified linear DNAs upon
            mammalian cell transfection
  JOURNAL   Unpublished
REFERENCE   2  (bases 1 to 6450)
  AUTHORS   Lim,S., Yocum,R.R., Silver,P.A. and Way,J.C.
  TITLE     Direct Submission
  JOURNAL   Submitted (22-DEC-2022) Systems Biology, Harvard Medical School,
            200 Longwood Ave, Warren Alpert Building Room 425, Silver Lab,
            Boston, MA 02115, USA
FEATURES             Location/Qualifiers
     source          1..6450
                     /organism="Vector PAS_122122"
                     /mol_type="other DNA"
                     /db_xref="taxon:3022699"
     gene            complement(145..1005)
                     /gene="blaTEM"
                     /allele="blaTEM-116"
     CDS             complement(145..1005)
                     /gene="blaTEM"
                     /allele="blaTEM-116"
                     /note="confers resistance to ampicillin, carbenicillin,
                     and related"
                     /codon_start=1
                     /transl_table=11
                     /product="broad-spectrum class A beta-lactamase TEM-116"
                     /protein_id="WBY69766.1"
                     /translation="MSIQHFRVALIPFFAAFCLPVFAHPETLVKVKDAEDQLGARVGY
                     IELDLNSGKILESFRPEERFPMMSTFKVLLCGAVLSRIDAGQEQLGRRIHYSQNDLVE
                     YSPVTEKHLTDGMTVRELCSAAITMSDNTAANLLLTTIGGPKELTAFLHNMGDHVTRL
                     DRWEPELNEAIPNDERDTTMPVAMATTLRKLLTGELLTLASRQQLIDWMEADKVAGPL
                     LRSALPAGWFIADKSGAGERGSRGIIAALGPDGKPSRIVVIYTTGSQATMDERNRQIA
                     EIGASLIKHW"
     regulatory      complement(1006..1110)
                     /regulatory_class="promoter"
                     /note="AmpR promoter"
     regulatory      1581..1960
                     /regulatory_class="enhancer"
                     /note="human cytomegalovirus immediate early enhancer"
     regulatory      1961..2164
                     /regulatory_class="promoter"
                     /note="human cytomegalovirus (CMV) immediate early
                     promoter"
     regulatory      2237..2246
                     /regulatory_class="other"
                     /note="Kozak sequence"
     CDS             2623..3111
                     /note="enhanced green fluorescent protein"
                     /codon_start=1
                     /transl_table=11
                     /product="eGFP"
                     /protein_id="WBY69767.1"
                     /translation="MKRHDFFKSAMPEGYVQERTIFFKDDGNYKTRAEVKFEGDTLVN
                     RIELKGIDFKEDGNILGHKLEYNYNSHNVYIMADKQKNGIKVNFKTRHNIEDGSVQLA
```

```
                     DHYQQNTPIGDGPVLLPDNHYLSTQSALSKDPNEKRDHMVLLEFVTAAGITHGMDELY
                     KS"
     regulatory      3221..3720
                     /regulatory_class="promoter"
                     /note="mouse phosphoglycerate kinase 1 promoter"
     CDS             3733..4332
                     /note="confers resistance to puromycin"
                     /codon_start=1
                     /transl_table=11
                     /product="puromycin N-acetyl-transferase"
                     /protein_id="WBY69768.1"
                     /translation="MTEYKPTVRLATRDDVPRAVRTLAAAFADYPATRHTVDPDRHIE
                     RVTELQELFLTRVGLDIGKVWVADDGAAVAVWTTPESVEAGAVFAEIGPRMAELSGSR
                     LAAQQQMEGLLAPHRPKEPAWFLATVGVSPDHQGKGLGSAVVLPGVEAAERAGVPAFL
                     ETSAPRNLPFYERLGFTVTADVEVPEGPRTWCMTRKPGA"
     misc_feature    4339..4927
                     /note="woodchuck hepatitis virus posttranscriptional
                     regulatory element"
     regulatory      5306..5427
                     /regulatory_class="polyA_signal_sequence"
                     /note="SV40 polyadenylation signal"
     regulatory      complement(5482..5512)
                     /regulatory_class="promoter"
                     /note="promoter for the E. coli lac operon"
     rep_origin      complement(5836..6424)
                     /note="high-copy-number ColE1/pMB1/pBR322/pUC origin of
                     replication"
                     /direction=LEFT
ORIGIN
        1 gtctgacgct cagtggaacg aaaactcacg ttaagggatt ttggtcatga gattatcaaa
       61 aaggatcttc acctagatcc ttttaaatta aaaatgaagt tttaaatcaa tctaaagtat
      121 atatgagtaa acttggtctg acagttacca atgcttaatc agtgaggcac ctatctcagc
      181 gatctgtcta tttcgttcat ccatagttgc ctgactcccc gtcgtgtaga taactacgat
      241 acgggagggc ttaccatctg gccccagtgc tgcaatgata ccgcgagacc cacgctcacc
      301 ggctccagat ttatcagcaa taaaccagcc agccggaagg gccgagcgca gaagtggtcc
      361 tgcaacttta tccgcctcca tccagtctat taattgttgc cgggaagcta gagtaagtag
      421 ttcgccagtt aatagtttgc gcaacgttgt tgccattgct acaggcatcg tggtgtcacg
      481 ctcgtcgttt ggtatggctt cattcagctc cggttcccaa cgatcaaggc gagttacatg
      541 atcccccatg ttgtgcaaaa aagcggttag ctccttcggt cctccgatcg ttgtcagaag
      601 taagttggcc gcagtgttat cactcatggt tatggcagca ctgcataatt ctcttactgt
      661 catgccatcc gtaagatgct tttctgtgac tggtgagtac tcaaccaagt cattctgaga
      721 atagtgtatg cggcgaccga gttgctcttg cccggcgtca atacgggata ataccgcgcc
      781 acatagcaga actttaaaag tgctcatcat tggaaaacgt tcttcggggc gaaaactctc
      841 aaggatctta ccgctgttga gatccagttc gatgtaaccc actcgtgcac ccaactgatc
      901 ttcagcatct tttactttca ccagcgtttc tgggtgagca aaaacaggaa ggcaaaatgc
      961 cgcaaaaaag ggaataaggg cgacacggaa atgttgaata ctcatactct tcctttttca
     1021 atattattga agcatttatc agggttattg tctcatgagc ggatacatat ttgaatgtat
     1081 ttagaaaaat aaacaaatag gggttccgcg cacatttccc cgaaaagtgc cacctgacgt
     1141 ctaagaaacc attattatca tgacattaac ctataaaaat aggcgtatca cgaggccctt
     1201 tcgtctcgcg cgtttcggtg atgacggtga aaacctctga cacatgcagc tcccggagac
     1261 ggtcacagct tgtctgtaag cggatgccgg gagcagacaa gcccgtcagg gcgcgtcagc
     1321 gggtgttggc gggtgtcggg gctggcttaa ctatgcggca tcagagcaga ttgtactgag
     1381 agtgcaccat atgcggtgtg aaataccgca cagatgcgta aggagaaaat accgcatcag
     1441 gcgccattcg ccattcaggc tgcgcaactg ttgggaaggg cgatcggtgc gggcctcttc
     1501 gctattacgc cagctggcga aagggggatg tgctgcaagg cgattaagtt gggtaaggtc
     1561 tcagggctga tatacgcgtt gacattgatt attgactagt tattaatagt aatcaattac
     1621 ggggtcatta gttcatagcc catatatgga gttccgcgtt acataactta cggtaaatgg
     1681 cccgcctggc tgaccgccca acgacccccg cccattgacg tcaataatga cgtatgttcc
     1741 catagtaacg ccaataggga ctttccattg acgtcaatgg gtggactatt tacggtaaac
     1801 tgcccacttg gcagtacatc aagtgtatca tatgccaagt acgcccccta ttgacgtcaa
     1861 tgacggtaaa tggcccgcct ggcattatgc ccagtacatg accttatggg actttcctac
     1921 ttggcagtac atctacgtat tagtcatcgc tattaccatg gtgatgcggt tttggcagta
     1981 catcaatggg cgtggatagc ggtttgactc acggggattt ccaagtctcc accccattga
```

2041 cgtcaatggg agtttgtttt ggcaccaaaa tcaacgggac tttccaaaat gtcgtaacaa
2101 ctccgcccca ttgacgcaaa tgggcggtag gcgtgtacgg tgggaggtct atataagcag
2161 agctctctgg ctaactagag aacccactgc ttagtcgatc ctaccatcca ctcgacacac
2221 ccgccagcgg ccgctcgcca ccatggtaag tgtgaatcga agcgcggctt cagaataccg
2281 ttttggctac aggatacaaa gcccatcgtg gtcctcagat atcgtgcacg tagagttgca
2341 ccgcacgcag tggctaactt ctctctttct ctctccctcc ctgtctttca ggctagcaaa
2401 ggagaagaac tcttcactgg agttgtccca attcttgttg aattagatgg tgatgttaac
2461 ggccacaagt tctctgtcag tggagagggt gaaggtgatg caacatacgg aaaacttacc
2521 ctgaagttca tctgcactac tggcaaactg cctgttccgt ggccgacact agtgacgacg
2581 ctctgctatg gcgtccagtg cttttcaaga tacccggatc acatgaaacg gcatgacttt
2641 ttcaagagtg ccatgcccga aggttatgta caggaaagga ccatcttctt caaagatgac
2701 ggcaactaca agacacgtgc tgaagtcaag tttgaaggtg atacccttgt taatagaatc
2761 gagttaaaag gtattgactt caaggaagat ggcaacattc tgggacacaa attggaatac
2821 aactataact cacacaatgt atacatcatg gcagacaaac aaaagaatgg aatcaaagtg
2881 aacttcaaga cccgccacaa cattgaagat ggaagcgttc aactagcaga ccattatcaa
2941 caaaatactc caattggcga tggccctgtc cttttaccag acaaccatta cctgtccaca
3001 caatctgccc tttcgaaaga tcccaacgaa aagagagatc acatggtcct tcttgagttt
3061 gtaacagctg ctgggattac acatggcatg gatgaactat acaaatcctg agttgtaaat
3121 gagcacacaa aatacacatg ctaaaatatt atattctatg acctttataa aatcaaccaa
3181 aatcttcttt ttaataactt tagtatcaat aattaattaa gggtagggga ggcgcttttc
3241 ccaaggcagt ctggagcatg cgctttagca gccccgctgg gcacttggcg ctacacaagt
3301 ggcctctggc ctcgcacaca ttccacatcc accggtaggc gccaaccggc tccgttcttt
3361 ggtggcccct tcgcgccacc ttctactcct cccctagtca ggaagttccc ccccgccccg
3421 cagctcgcgt cgtgcaggac gtgacaaatg gaagtagcac gtctcactag tctcgtgcag
3481 atggacagca ccgctgagca atggaagcgg gtaggccttt ggggcagcgg ccaatagcag
3541 ctttgctcct tcgctttctg ggctcagagg ctgggaaggg gtgggtccgg gggcgggctc
3601 aggggcgggc tcaggggcgg ggcgggcgcc cgaaggtcct ccggaggccc ggcattctgc
3661 acgcttcaaa agcgcacgtc tgccgcgctg ttctcctctt cctcatctcc gggcctttcg
3721 gatatcgcca ccatgaccga gtacaagccc accgtgcgac tggcaacacg agacgatgta
3781 cccagagcag taagaacgct cgctgctgcg ttcgccgact acccagctac acggcacaca
3841 gttgacccag accgacatat tgaacgggtc accgaactcc aggagctttt tctcactcgc
3901 gtggggctcg atatcggtaa agtttgggtg gccgacgatg gagccgcggt tgcagtgtgg
3961 acaacaccgg aatcagttga agccggtgcg gtttttgcgg aaattggccc acgaatggcg
4021 gaactttccg gatcccgctt ggccgcgcag cagcagatgg agggacttct tgctccgcat
4081 aggccgaaag aaccagcttg gttccttgcc acagttggtg tgtcacctga tcatcaaggc
4141 aaggggctgg ggtctgccgt tgttctgcca ggcgtggagg ccgcggaaag ggctggggtg
4201 cctgccttct tggagacttc agctccacga aatctcccgt tttatgaacg cctgggattt
4261 actgttaccg ctgatgtgga ggttcctgaa ggtcctcgaa cttggtgtat gactaggaaa
4321 ccaggcgcct gagtcgacaa tcaacctctg gattacaaaa tttgtgaaag attgactggt
4381 attcttaact atgttgctcc ttttacgcta tgtggatacg ctgctttaat gcctttgtat
4441 catgctattg cttcccgtat ggctttcatt ttctcctcct tgtataaatc ctggttgctg
4501 tctctttatg aggagttgtg gcccgttgtc aggcaacgtg gcgtggtgtg cactgtgttt
4561 gctgacgcaa cccccactgg ttggggcatt gccaccacct gtcagctcct ttccgggact
4621 ttcgctttcc ccctccctat tgccacggcg gaactcatcg ccgcctgcct tgcccgctgc
4681 tggacagggg ctcggctgtt gggcactgac aattccgtgg tgttgtcggg gaaatcatcg
4741 tcctttcctt ggctgctcgc ctgtgttgcc acctggattc tgcgcgggac gtccttctgc
4801 tacgtccctt cggccctcaa tccagcggac cttccttccc gcggcctgct gccggctctg
4861 cggcctcttc cgcgtcttcg ccttcgccct cagacgagtc ggatctccct ttgggccgcc
4921 tccccgcctg gtacctttaa gaccaatgac ttacaaggca gctgtagatc ttagccactt
4981 tttaaaagaa aaggggggac tggaagggct aattcactcc caacgaagat aagatctgct
5041 ttttgcttgt actgggactc tctggttaga ccagatctga gcctgggagc tctctggcta
5101 actagggaac ccactgctta agcctcaata aagcttgcct tgagtgcttc aagtagtgtg
5161 tgcccgtctg ttgtgtgact ctggtaacta gagatccctc agaccctttt agtcagtgtg
5221 gaaaatctct agcagtagta gttcatgtca tcttattatt cagtatttat aacttgcaaa
5281 gaaatgaata tcagagagtg agaggaactt gtttattgca gcttataatg gttacaaata
5341 aagcaatagc atcacaaatt tcacaaataa agcatttttt tcactgcatt ctagttgtgg
5401 tttgtccaaa ctcatcaatg tatcttatca tgtctggctc tagctatccc gagagaccct
5461 gtatccgctc acaattccac acaacatacg agccggaagc ataaagtgta aagcctgggg
5521 tgcctaatga gtgagctaac tcacattaat tgcgttgcgc tcactgcccg ctttccagtc
5581 gggaaacctg tcgtgccagc tgcattaatg aatcggccaa cgcgcgggga gaggcggttt
5641 gcgtattggg cgctcttccg cttcctcgct cactgactcg ctgcgctcgg tcgttcggct
5701 gcggcgagcg gtatcagctc actcaaaggc ggtaatacgg ttatccacag aatcagggga
5761 taacgcagga aagaacatgt gagcaaaagg ccagcaaaag gccaggaacc gtaaaaaggc

```
     5821 cgcgttgctg gcgtttttcc ataggctccg cccccctgac gagcatcaca aaaatcgacg
     5881 ctcaagtcag aggtggcgaa acccgacagg actataaaga taccaggcgt ttccccctgg
     5941 aagctccctc gtgcgctctc ctgttccgac cctgccgctt accggatacc tgtccgcctt
     6001 tctcccttcg ggaagcgtgg cgctttctca tagctcacgc tgtaggtatc tcagttcggt
     6061 gtaggtcgtt cgctccaagc tgggctgtgt gcacgaaccc cccgttcagc ccgaccgctg
     6121 cgccttatcc ggtaactatc gtcttgagtc caacccggta agacacgact tatcgccact
     6181 ggcagcagcc actggtaaca ggattagcag agcgaggtat gtaggcggtg ctacagagtt
     6241 cttgaagtgg tggcctaact acggctacac tagaaggaca gtatttggta tctgcgctct
     6301 gctgaagcca gttaccttcg gaaaaagagt tggtagctct tgatccggca aacaaaccac
     6361 cgctggtagc ggtggttttt ttgtttgcaa gcagcagatt acgcgcagaa aaaaaggatc
     6421 tcaagaagat cctttgatct tttctacggg
//
```

Figure S4. The sequence of the plasmid PAS_122122 (Figure 1A), represented in GenBank format.

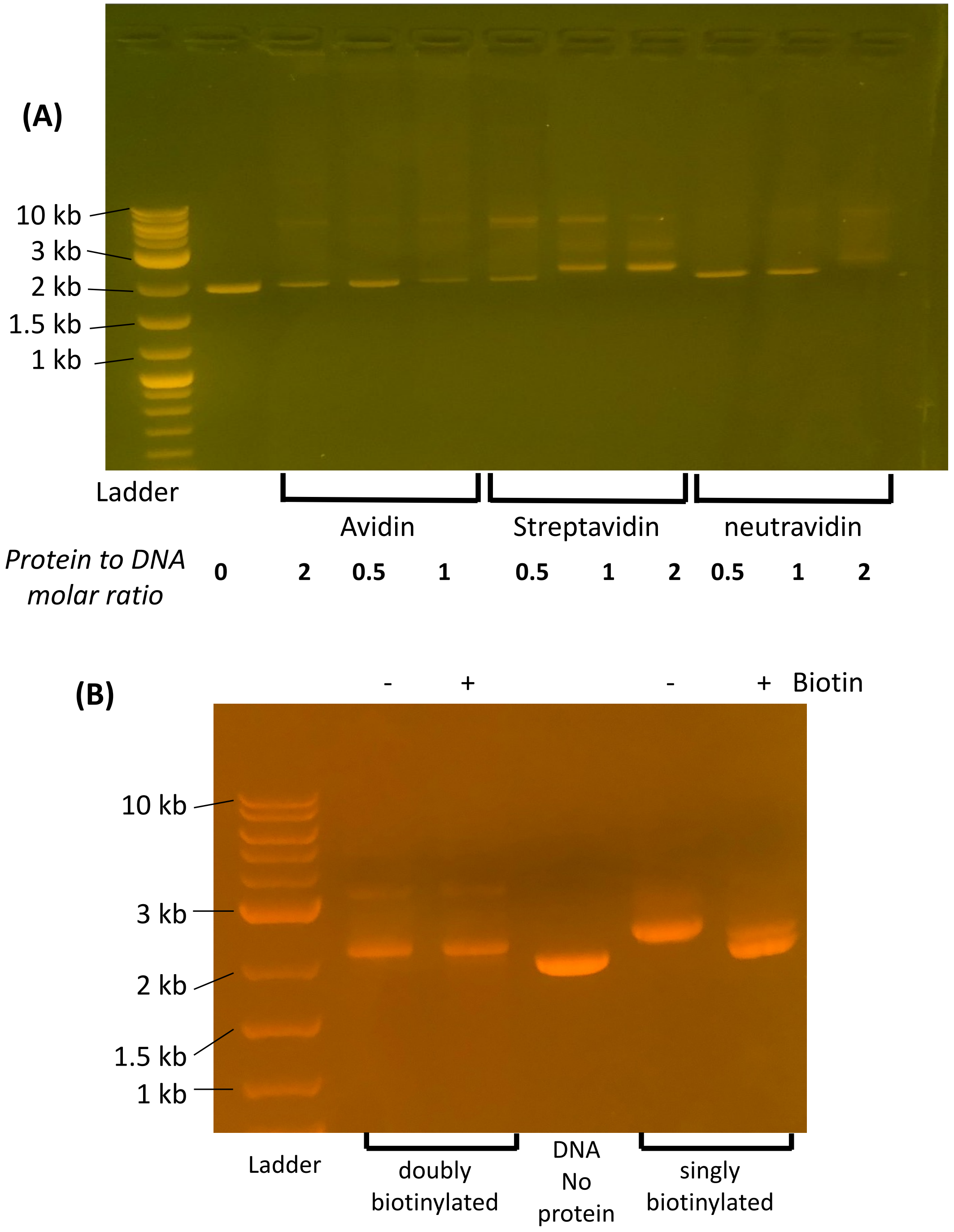

Figure S5. The agarose gels of Figure 2, showing the full gel pictures without cropping and with molecular weight standards. **(A)** This figure corresponds to Figure 2B. **(B)** This figure corresponds to Figure 2C.

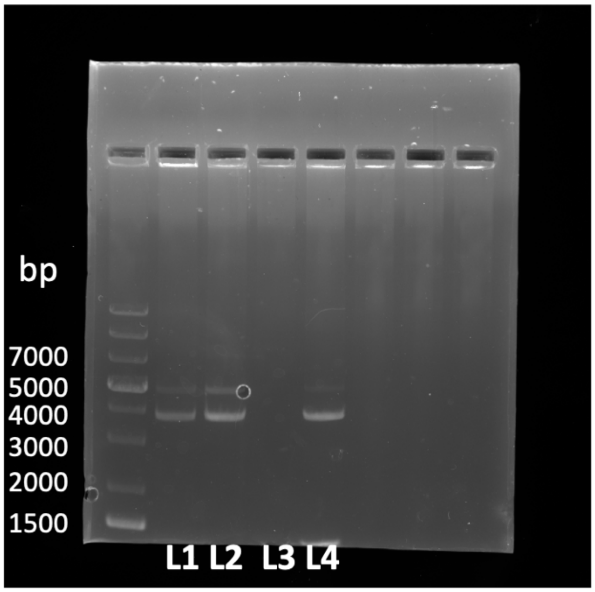

Figure S6. The agarose gels showing the exonuclease resistance of synthesized CELiD. CELiD and unmodified linear DNA were digested with Exo III nuclease for 30 min at 37°C. Each lane corresponds to the following. L1: digested CELiD; L2: non-digested control CELiD; L3: digested unmodified linear DNA; L4: non-digested control linear DNA. We hypothesize that the amount of DNA in lane L1 is less than in lane L2 because the sample was freeze-thawed and some of the DNA became nicked allowing for Exo III digestion.

Supplementary analysis: Calculations and estimates on copy numbers of functional DNA during transfection, and on the physical size of transfecting DNA.

The relative rate of DNA integration estimated in Figure 3 may also be normalized to the copies of input DNA. In the transfections of Figure 3, we used 50 ng of DNA to transfect about 50,000 cells, or about 1 pg/cell. The molecular weight of the entire plasmid is about $4.25*10^6$ Daltons (6,450 bp * 660 Daltons/bp). Thus, 1 pg of intact plasmid consists of about $1.4*10^5$ molecules. A similar calculation indicates that 1 pg of the linear fragments consists of about $1.8*10^5$ molecules. Based on the data in Figure 3, the frequency of DNA integration can be estimated at 1 integration per $2.8*10^7$ input plasmids, and for the various types of linear DNAs, the frequency is about 1 integration per $1.8*10^6$ DNAs.

Wang et al. [1] performed a complementary study of the mammalian cell transfection process, in which they quantitated DNA entering cells microscopically. They found that in many cells, tens of thousands of plasmids form a cluster in the cytoplasm that is actively segregated to one daughter cell. The presence of such clusters is not correlated with expression of an encoded fluorescent protein, so the cytoplasmic clusters appear to be non-functional. The number of plasmids in such a cluster is comparable to the number of plasmids per cell in a typical transfection, as we calculated above. In addition, Wang et al. estimate that this cytoplasmic population contains about 100- to 1000-fold more DNA than seen in the clusters of plasmids in the nucleus, whose presence is correlated with gene expression. This corresponds roughly to our estimate of about 100 plasmids per cell that contribute to functional gene expression, and independently validates our estimate.

The plasmid, linear, CELiD, and blocked-end linear DNAs show significantly different transfection frequencies (Figure 3, Supplementary Figure S3), with supercoiled plasmids being more efficient at transient transfection. These differences may result from differences in supercoiling itself, in the compactness of the DNA, or in the presence of free ends that may be sites of nuclease attack. We note that since the persistence length of linear DNA is about 200 base pairs and the length of our linear DNAs is about 3900 bp, if the DNA forms a random coil its end-to-end length would be about 0.3 microns. This is calculated by saying that the DNA undergoes a random walk of 19.5 steps that on average gives an end-to-end distance roughly equal to the square root of the number of steps (With each step being 200 bp (68 nanometers), the result of this random walk is $(19.5)^{1/2} * 68 = 300$ nanometers. An effective length of 0.3 microns is fairly large compared to the diameter of a mammalian cell. Supercoiled DNA is presumed to be more compact.

Reference

1. Wang, X., Le, N., Denoth-Lippuner, A., Kroschewski, R. Asymmetric partitioning of transfected DNA during mammalian cell division. Proc. Natl. Acad. Sci. U. S. A. 113, 7177-7182 (2016).
